# Soluble endoglin reflects endothelial dysfunction in myocardial infarction patients: a retrospective observational study

**DOI:** 10.7150/ijms.115222

**Published:** 2025-07-04

**Authors:** J. Urbankova Rathouska, J. Mrazkova, C. Andrys, K. Jankovicova, K. Tripska, P. Fikrova, I. Nemeckova, S. Eissazadeh, P. Wohlfahrt, J. Pitha, P. Nachtigal

**Affiliations:** 1 Department of Biological and Medical Sciences, Faculty of Pharmacy in Hradec Králové, Charles University in Prague, Czech Republic.; 2 Cardiology Department, Cardiac Centre, Institute for Clinical and Experimental Medicine, Prague, Czech Republic.; 3 Department of Immunology and Allergology, University Hospital Hradec Králové and Charles University, Faculty of Medicine in Hradec Králové, Czech Republic.; 4 Department of PISA, Cardiac Centre, Institute for Clinical and Experimental Medicine, Prague, Czech Republic.

**Keywords:** myocardial infarction, soluble endoglin, soluble endocan.

## Abstract

Acute manifestations of ischemic heart disease are among the most serious and fatal consequences of atherosclerotic processes. In this study, we hypothesized that a soluble proprotein convertase subtilisin/kexin type 9 (PCSK9), soluble bone morphogenetic protein 4 (BMP-4), soluble E-selectin (sE-selectin), soluble endoglin (sENG) and soluble endocan (Endocan) would differ from healthy controls in myocardial infarction (MI) patients admitted to the hospital without any previous history of cardiovascular disease and with no cardioprotective drugs taken before admission. The study was conducted using a cross-sectional design.

We analyzed data from 79 patients (mean age 54.1 ± 8.9, 18% of women) admitted for the first manifestation of MI and with no history of cardioprotective treatment use before the event. As a control group, we analyzed 17 age-matched healthy volunteers (mean age 51.5 ± 8.6, 47% of women). In addition to routinely obtaining clinical and laboratory data, we analyzed plasma concentrations of the aforementioned biomarkers using ELISA and Luminex analyses.

Patients with MI did not differ from healthy controls in total cholesterol, LDL, non-HDL, and triglyceride levels. PCSK9, BMP-4, and sE-selectin levels did not differ significantly between the MI and the control group. Patients with MI had significantly higher sENG and Endocan levels than the control group. In addition, levels of sENG were significantly higher in patients with higher body mass index (BMI) and in smokers.

We demonstrated that sENG could serve as a biomarker reflecting endothelial dysfunction in MI patients without prior treatment for cardiovascular risk factors.

## Introduction

Worldwide, cardiovascular diseases continue to place a heavy load on overall mortality, with acute coronary syndrome frequently being the first symptom to manifest 1. This occurs due to the rupture of atherosclerotic plaques within the coronary arteries, leading to myocardial ischemia, necrosis, and subsequent clinical presentations. Thus, early diagnosis and risk assessment are essential for directing prompt therapies and enhancing patient outcomes 2.

The endothelium plays an essential role in coronary artery disease. In the presence of cardiovascular risk factors like hypertension, hyperlipidemia, or hyperglycemia, it changes its quiescence towards the activated state with expression of adhesion molecules and inflammatory markers that alter the permeability and vasoactive properties of the endothelium 3. For this reason, endothelial dysfunction (ED) has been strongly associated with the increased risk of myocardial infarction (MI) 3,4.

Several markers of ED have been studied in relation to endothelial dysfunction, with some shown to be more or less specific in the context of various vascular pathologies. However, currently, there is no “golden standard” circulating biomarker for coronary artery disease 3. We introduce possibly novel and interesting endothelial dysfunction and inflammation biomarkers related to therapy-naïve individuals with no symptomatic cardiovascular disease suffering MI episodes.

Proprotein convertase subtilisin/kexin type 9 is a critical player in cholesterol metabolism related to endothelial dysfunction, inflammation, and atherosclerosis 5. Moreover, it was proposed as an adverse factor for cardiovascular risk beyond dyslipidemia 6. Bone morphogenetic proteins (BMPs) are members of the Transforming Growth Factor β (TGFβ) superfamily, whereby BMP-4 is evidenced as a critical regulator in vascular biology. Its crucial role in leukocyte recruitment during vascular inflammation 7, as well as its potential involvement in hypertension development, have been demonstrated 8. Several adhesion molecules, including E-selectin, are expressed during the inflammatory response of vascular endothelium. Moreover, studies report increased levels of sE-selectin as circulatory markers reflecting the burden of coronary artery disease 3.

Endoglin (ENG, CD105) is a 180 kDa transmembrane glycoprotein considered a co-receptor for ligands of the TGFβ superfamily 9. There are two different isoforms of membrane ENG expressed by various cells (endothelial cells, macrophages, smooth muscle cells) and soluble endoglin (sENG) circulating in plasma or cell culture medium 10. sENG is the N-terminal cleavage product of the extracellular domain of ENG formed by the activity of matrix metalloproteinases 11-13 that is released into the circulation. In addition to matrix metalloproteinases, thrombin has recently been described to generate sEng 14. sENG can be detected and used as a biomarker in various cardiovascular and metabolic disorders, such as preeclampsia 15, hypercholesterolemia 16, familial hypercholesterolemia 17, atherosclerosis 18, arterial hypertension 19, septic shock disease 20, and liver alterations 21. Moreover, based on our experimental data, increased sENG levels were demonstrated in the development of early aortic endothelial dysfunction 22 and liver sinusoidal endothelial dysfunction during NASH progression 21 and correlated with total cholesterol levels and progression of atherosclerosis *in vivo* 23, suggesting their importance in the development of endothelial dysfunction. In addition, we demonstrated that high levels of sENG aggravate aortic endothelial dysfunction 24 and endothelial inflammation 25, suggesting their potentially harmful effects on vascular endothelium.

However, sENG level changes in acute coronary syndrome (ACS) and coronary artery disease (CAD) show controversial data. For instance, Cruz-Gonzalez et al. found sENG levels in acute myocardial infarction patients to be lower than those of healthy individuals 26. On the contrary, sENG levels were higher in patients with ruptured plaque and unstable plaque than those with stable plaque, suggesting that sENG reflects the plaque stability and risk of acute coronary syndrome 27.

Endocan, or Endothelial Cell-Specific Molecule-1 (ESM-1), a dermatan sulfate proteoglycan, is expressed primarily by the vascular endothelium and has been found freely circulating in the bloodstream as a soluble endocan (Endocan) 28. Indeed, elevated plasma levels of Endocan may reflect endothelial activation and dysfunction 29. Moreover, Endocan was shown to be an independent predictor of heart failure 30, a surrogate marker for hypertension 31, coronary artery disease, coronary slow flow 32, angina, and subclinical atherosclerosis 33.

Our previous extensive experimental studies, as well as those of others, have indicated a strong potential negative impact of high sENG levels on endothelial dysfunction development 24 and liver metabolism 34. In this study, we focused on the human population. Specifically, we addressed whether there were any differences in the above-mentioned biomarkers of endothelial dysfunction between patients with MI and controls. In addition, we analyzed the potential association of these biomarkers with traditional lipid risk factors and smoking habits.

## Materials and Methods

Data from patients recruited for the AMBITION (Institute for Clinical and Experimental Medicine Acute Myocardial Infarction Registry) registry were analyzed. This registry collects clinical and laboratory data from all consecutive patients hospitalized for MI at a tertiary heart center since June 2017 35. This observational study was approved by the ethical committee of Faculty Thomayer Hospital and Institute for Clinical and Experimental Medicine, Prague, Czech Republic, on June 13, 2018, under registration number G-18-52. The data from this project were accessed and analyzed (blinded for any personal information of participants) from 20.11.2023 to 14.5.2024, together with measuring soluble endoglin and other factors from available frozen blood samples. The methodology of this study has been previously described 36. Briefly, in the hospital, all patients underwent detailed interviews regarding their health status, with additional information obtained from medical records and laboratory studies. Definitions of cardiovascular risk factors already present before MI were as follows: patients were considered smokers if they reported smoking at least one cigarette per day 12 months before developing MI; a history of diabetes was defined as the use of oral antidiabetic drugs or insulin at the time of hospital admission (for this study, patients treated by drugs including insulin were excluded); arterial hypertension was defined as self-reported hypertension and/or use of antihypertensive medications upon admission (for this study, patients treated by drugs were excluded). The purpose of this study is to examine persons with a history of diabetes and hypertension without any drug treatment. The Institutional Review Board of the Prague-based Institute for Clinical and Experimental Medicine approved the study, and all participants signed informed consent. The investigation conformed to the principles outlined in the Declaration of Helsinki. The exclusion criteria were unwillingness to sign informed consent and age above 65.

The project was a retrospective study involving plasma samples from 79 patients admitted with MI with neither a previous history of cardiovascular disease nor a history of treatment by cardioprotective drugs. The patients were hospitalized at the Department of Cardiology at the Institute for Clinical and Experimental Medicine (Prague, Czech Republic). The control group comprised samples from 17 age-matched and healthy volunteers of both genders recruited from preventive medical inspections to assess differences in the study parameters. Clinical data of both groups, including age, sex, diabetes mellitus, hypertension, and smoking habits, were collected. The study protocol was approved by the local institutional review board, and informed consent was obtained from all patients. The clinical characteristics of both groups of subjects in the study are summarized in Table 1.

### Blood samples and analyses

All patients' blood for laboratory analyses was collected in the morning after admission in fasting status. Lipid parameters were analyzed using a fully automated enzymatic method (COBAS MIRA S analyzer, Roche Diagnostics, Basel, Switzerland) with enzymatic kits produced by the same manufacturer. For this study, only lipid parameters were used as follows: total cholesterol, LDL, HDL, triglycerides, and non-HDL cholesterol.

For this study, data from patients under 65 years old who were hospitalized for MI between 2020 and 2023 were analyzed. The Institutional Review Board of the Prague-based Institute for Clinical and Experimental Medicine approved the study, and all participants signed informed consent. The investigation conformed to the principles outlined in the Declaration of Helsinki. The exclusion criteria were unwillingness to sign informed consent and age above 65. Blood samples were collected the morning after hospital admission with MI or during regular medical inspections in case of healthy controls. The blood was taken in EDTA-containing tubes and centrifuged within 30 minutes at 1500G for 15 min at room temperature. Plasma samples were aliquoted and stored at -80°C before the biochemical and proteomic analysis.

### Analysis of biomarkers of endothelial dysfunction

The concentrations of soluble endoglin (CD105) were assessed in plasma samples by sandwich enzyme-linked immunosorbent assay technique (ELISA) using the Quantikine Human Endoglin/CD105 ELISA kit (R&D Systems, MN, USA) according to the manufacturer's instructions. Samples were undiluted. The sensitivity of the kit was 0.007 ng/mL. The absorbance values were measured at 450 nm with a Multiskan RC ELISA reader (Thermo Fisher Scientific, MA, USA). The levels of Endocan, PCSK9, BMP-4, and sE-selectin were determined using the Human Premixed Multi-Analyte Magnetic Luminex Kit (R&D Systems, MN, USA), according to the manufacturer's protocol.

### Statistical analysis

The statistical analysis was performed using GraphPad Prism software version 9.2 (GraphPad Software Inc., San Diego, CA, USA). Clinical data are expressed as mean** ±** standard deviation (SD). Laboratory data of endothelial dysfunction markers are presented as median with interquartile range (IQR). In the first place, a normality test was performed in all data, and based on these, non-parametric tests were used for further analyses. Direct group-group comparisons were carried out using a non-parametric Mann-Whitney test. Correlations were determined using Spearman´s r and p-value. Linear regression analyses were determined using the Beta coefficient, 95% CI, and p-value. P-values < 0.05 were considered statistically significant.

## Results

### Patients with MI did not differ from healthy controls in parameters of total cholesterol, LDL, triglycerides, and non-HDL levels

As shown in Table 1, there was a significantly lower representation of women and a higher representation of smokers in the MI group. Analysis of lipid parameters revealed no differences between MI patients and controls on the level of total cholesterol, LDL, non-HDL cholesterol, and triglycerides in plasma. Both groups also did not differ in BMI values. In contrast, the group of MI patients had significantly decreased levels of HDL compared to controls.

### Patients with MI had significantly increased levels of sENG and Endocan in plasma compared to controls

**Figure 1:**
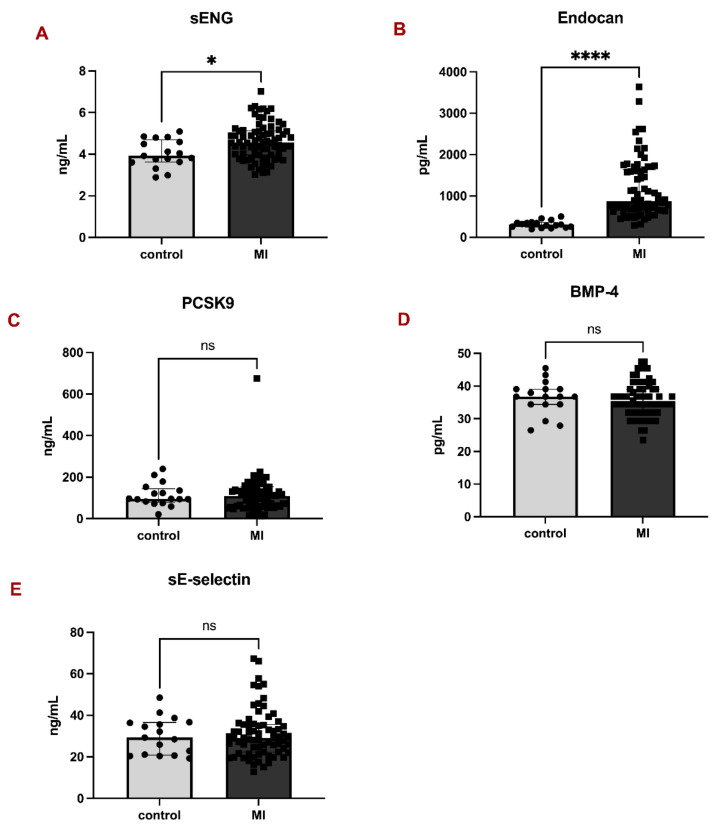
Parameters of endothelial dysfunction in controls and patients with myocardial infarction. Bar graphs representing the levels of sENG (A), Endocan (B), PCSK9 (C), BMP-4 (D), and sE-selectin (E) between controls and MI patients. All data are shown as median with interquartile range. Mann-Whitney test, *p < 0.05, ****p < 0.0001.

Analysis of plasma markers revealed significantly higher levels of sENG in MI patients compared to controls: 4.56 (3.97-5.12) vs 3.92 (3.62-4.69), p = 0.0113 (Fig. 1A). Similarly, the difference between MI patients and controls in Endocan levels proved significant: 868.4 (654.4-1631.0) vs 308.3 (245.1-358.0), p < 0.0001 (Fig. 1B). On the contrary, there were no significant differences in the levels of PCSK9 (Fig. 1C), BMP-4 (Fig. 1D), and sE-selectin (Fig. 1E) between the two groups.

### sENG levels were significantly higher in BMI at risk and smoking MI patients

**Figure 2:**
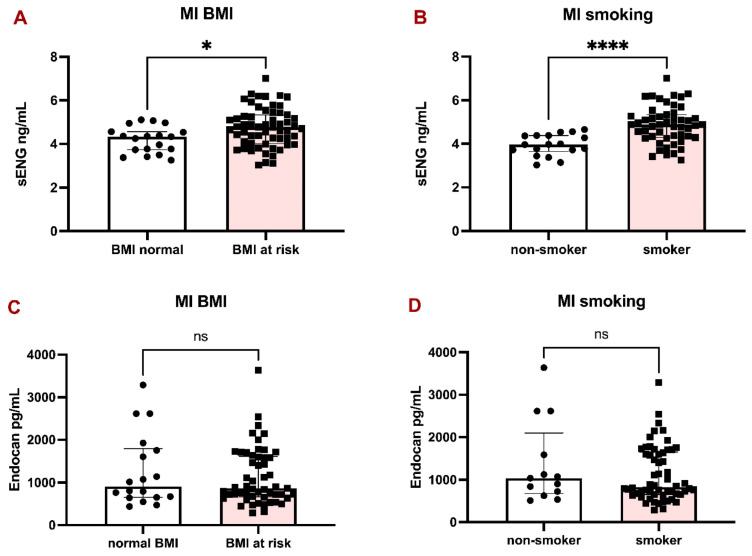
sENG and Endocan levels in the plasma of MI patients with respect to BMI and smoking habits. Bar graphs representing the levels of sENG in MI patients according to BMI (A) and smoking habits (B). Bar graphs representing the levels of Endocan in MI patients according to BMI (C) and smoking habits (D). All data are shown as median with interquartile range. Mann-Whitney test, *p < 0.05, ****p < 0.0001.

The patients with MI were divided into two groups according to BMI (over 25 kg/m^2^ considered at risk) and smoking habits. sENG was significantly higher in patients with BMI at risk (Fig. 2A) and in smokers (Fig. 2B), compared to Endocan, which did not significantly differ between BMI groups (Fig. 2C) and in smokers (Fig. 2D).

Since there was a considerably higher representation of smokers in the MI group (Table 1), we decided to perform further evaluation. A simple linear regression analysis initially showed that patients with MI had significantly higher plasma sENG levels compared to non-MI individuals (β = 0.59 ng/mL, 95% CI [0.15, 1.03], p = 0.009). However, given that smoking is a known risk factor for both MI and endothelial dysfunction, we conducted a multiple linear regression analysis adjusting for smoking status.

After adjustment for smoking, the association between MI and sENG levels was no longer statistically significant (β = 0.25 ng/mL, 95% CI [-0.19, 0.69], p = 0.256), whereas smoking remained a strong independent predictor of sENG levels (β = 0.71 ng/mL, 95% CI [0.37, 1.05], p <0.0001). The adjusted model explained 22% of the variance in sENG levels (R² = 0.22), compared to 7% (R² = 0.07) in the unadjusted model.

### LDL levels gave only a weak correlation with sENG and Endocan levels in the cohort of MI patients

The last evaluation searched for possible correlations between sENG and Endocan and traditional risk factors for MI. We found no correlation of total cholesterol with sENG (r=0.01902; P=0.8687) or with Endocan (r=0.1974; P=0.0989), no correlation of triglycerides with sENG (r= -0.03969; P=0.7301) or with Endocan (r=0.06089; P=0.6139), no correlation of HDL with sENG (r= -0.06275; P=0.5852) or with Endocan (r=0.03664; P=0.7616) and no correlation of non-HDL with sENG (r=0.1785; P=0.1179) or with Endocan (r=0.1770; P=0.1398).

**Figure 3:**
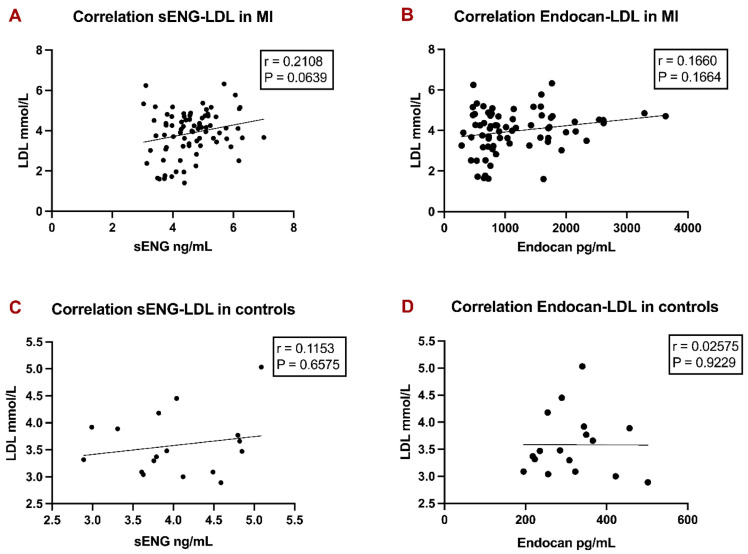
Correlation analyses of LDL with sENG and Endocan levels in MI patients and controls. Linear regression graphs of LDL with sENG in MI patients (A), LDL with Endocan in MI patients (B), LDL with sENG in controls (C), and LDL with Endocan in controls (D) were designed. Spearman r values were defined. P-values < 0.05 were considered statistically significant.

We found only a weak, non-significant correlation between LDL levels with sENG (Fig. 3A) in MI patients and LDL levels with Endocan (Fig. 3B) in MI patients. Both correlations also turned insignificant in controls (Fig. 3C, 3D). Data is presented as correlation coefficients and p-values in Figure 3.

## Discussion

In our study, we demonstrated for the first time that plasma sENG levels were substantially higher in survivors of MI than in healthy controls. Moreover, in the survivors, sENG levels were higher in smokers than in non-smokers, indicating an association of sENG with this robust risk factor of endothelial dysfunction and indirect evidence that sENG might be an important marker for severe vascular damage.

Despite important scientific advances, MI remains at the top of death causes worldwide 1. Searching for highly sensitive predictive indicators of these pathologies is, therefore, of great importance. Markers of endothelial dysfunction seem to be reasonable in evaluating the burden of coronary artery disease 3, and might bring an early diagnostic and prognostic tool for MI 2. Some already established and novel markers of ED have been studied. However, we specifically focused on the levels of sENG, Endocan, PCSK9, BMP-4, and sE-selectin.

In this study, we hypothesized that the above-mentioned circulating biomarkers of endothelial dysfunction would differ in patients admitted for the first myocardial infarction compared to healthy controls. We included a specific group of patients with MI with no previous symptomatic cardiovascular disease and with no cardiovascular therapy before the coronary event. The reason for selecting this specific cohort was based on the assumption that several groups of drugs, being frequently taken in cardio-metabolic disorders, might significantly modify the values of circulating ED markers and the state of the endothelium due to their pleiotropic effects 37,38. Accordingly, these patients were compared to a group of healthy volunteers who were not undergoing any cardio-metabolic treatment.

There were no differences in total cholesterol, triglycerides, LDL, and non-HDL levels between the MI and the control group. In contrast, there was a significant difference in HDL levels. This phenomenon may be partially attributed to the generally suboptimal values observed in patients with MI and the higher proportion of women in the control group. In general, these results indicate that the main atherogenic lipid parameters might not be sensitive enough to predict MI in patients with no previous history of cardiovascular events.

In the initial comparison, only sENG and Endocan reached statistical significance between the MI group and the control group. Notably, several papers have focused on the role of sENG in MI with discrepant observations 39-42. Interestingly, sENG may not be only a potential circulating biomarker of MI. It was demonstrated that high levels of sENG could aggravate endothelial dysfunction 22 and induce inflammatory markers expression 25. This suggests that high levels of sENG might even promote the progression or manifestation of MI by aggravating endothelial dysfunction and inflammation 43. It is also of interest to mention that sENG regulates the expression of angiogenesis-related proteins, leading to anti-angiogenic effects 44. In addition, elevated levels of sEng were associated with dysregulated angiogenesis and vascular remodeling in the brain 45. Indeed, vascular remodeling, including neoangiogenesis, occurs following the acute stage of myocardial infarction 46. Therefore, we propose that elevated levels of sENG may influence this angiogenic process in the heart, an aspect that warrants investigation in future prospective studies.

Endocan is a biomarker considered a potential prognostic tool for coronary artery disease 2. A meta-analysis found that Endocan levels in patients with coronary artery disease were higher than in the control group, suggesting its potential role as a marker reflecting atherosclerosis progression 47. Interestingly, similarly to sENG, Endocan is not only a biomarker of MI. It is widely accepted that Endocan is involved in the development of atherosclerosis, primarily by contributing to endothelial dysfunction 29. Also, data from this study indicate a possible benefit of Endocan levels evaluation in therapy-naïve patients, reflecting endothelial dysfunction in the risk group.

Our study also assessed the relationship between BMI at risk, smoking habits, and sENG and Endocan levels in MI patients. The analyses revealed that only sENG levels, but not Endocan levels, were significantly higher in patients with a BMI over 25 kg/m^2^, and an even more pronounced increase was found in smokers. It should be emphasized that our MI group included a significantly higher number of smokers compared to the control group. Our multivariable analysis also proved smoking as an independent predictor of high sENG levels. This is an interesting finding if we consider the contribution of smoking to endothelial dysfunction, upregulation of matrix metalloproteinases (MMPs) and plaque instability 48, and the role of MMPs in endoglin shedding and elevated sENG levels 12,49.

We also found a weak, non-significant correlation of sENG with LDL, indicating that sENG is relatively independent of LDL in MI patients. This is in line with the paper showing no correlation between LDL and sENG in patients with already established chronic coronary artery disease undergoing cardiac catheterization 50.

### Strengths and limitations of the study

The strength of our study lies in the robust experimental data that strongly support the biological plausibility of our findings in a well-defined patient group, indicating that sENG may also have the potential to cause vascular damage in humans.

Nevertheless, we are fully aware of the limitations, including the retrospective/cross-sectional design of this study, which does not allow us to reliably establish cause-and-effect relationships. The next limitation is that the samples for sENG measurements were collected during the acute phase of MI and may have been influenced by the acute pathophysiological changes occurring during this period. Another limitation is that we did not have a completely matched control group in this study. However, the main atherogenic lipid parameters were not different between the patients and the control group, and HDL cholesterol is recently not considered an anti-atherogenic lipid factor 51.

An important limitation of the study might be the gender distribution within the MI and control groups and its potential impact on the relevance of sENG levels. However, we took this aspect into account from the very beginning. Initially, we performed a statistical analysis, which showed no significant differences in sENG levels between men and women, either in the MI group or in the control group. In addition, the difference in sENG levels between MI patients and controls remained statistically significant when analyzing men and women separately. Therefore, we chose to include both sexes in the MI and control groups together to strengthen the statistical power and the biological plausibility of our findings. We can infer that the elevated endoglin concentrations observed in patients with MI highlight its significance as a marker of increased cardiovascular metabolic risk.

## Conclusion

For the first time in patients, we demonstrate that sENG may serve as a biomarker of endothelial dysfunction in MI patients who have not received prior treatment for cardiovascular risk factors. Prospective studies are needed to confirm the value of monitoring sENG levels in the prevention and management of endothelial dysfunction-related diseases.

## Figures and Tables

**Table 1:** Clinical data of controls and myocardial infarction patients

	Controls (n=17)	MI patients (n=79)	p
Age	51.5 ±8.6	54.1 ±8.9	0.1599
Women (%)	47	18	0.0142^*^
Smoking status (%)	18	65	0.0005^***^
Diabetes mellitus (%)	0	8.9	0.3464
Hypertension (%)	18	8.9	0.3753
Total cholesterol (mmol/L)	5.6 ±0.7	5.5 ±1.1	0.6123
Triglycerides (mmol/L)	1.2 ±0.3	1.6 ±1.2	0.0985
LDL (mmol/L)	3.6 ±0.6	3.9 ±1.1	0.0836
HDL (mmol/L)	1.6 ±0.4	1.2 ±0.4	<0.0001^****^
non-HDL (mmol/L)	4.0 ±0.6	4.3 ±1.2	0.1477
BMI	28.2 ±4.4	26.6 ±3.6	0.1116

Values are means ± SD, significant values marked with asterisks, abbreviations: MI - myocardial infarction patients, LDL - low-density lipoproteins, HDL - high-density lipoproteins, BMI - body mass index.
